# Supplementary material for: A systematic review of behavioral interventions to improve maternal outcomes for women in the United States at high risk for adverse pregnancy outcomes
Source: medRxiv. 2025 Jul 21:2025.07.18.25331742. Preprint. [Version 1] doi: 10.1101/2025.07.18.25331742 (PMC12330428; doi:10.1101/2025.07.18.25331742)
Supplement: Supplement 2 [file media-2.pdf]

First author, year of publication, (citation)	Title	Aim of Study	Number of participants	Length of intervention	When intervention ended	When monitoring participants ended	High risk feature trial was designed for	Intervention type	Outcome of intervention	Method of Recruitment	Race and ethnicity reported as separate categories	Notes on race and ethnicity reporting	Excluded women with pre-existing CVD factors other than feature trial was designed for	BMI requirement
Allman 2021	Markers of Branched-Chain Amino Acid Catabolism are Not Affected by Exercise Training in Pregnant Women with Obesity	Determine the effect of a moderate-intensity exercise intervention during pregnancy on maternal circulating branched-chain amino acids (BCAAs) and markers of BCAA catabolism, and their associations with insulin resistance.	80	~24 weeks	prenatal	prenatal	Overweight/obesity	Physical activity	Physical activity was safe for pregnant participants (intervention feasible)	Clinic	Yes	Race: Caucasian, African American, More than One/Missing	yes	obese
Basu 2021	Dietary Blueberry and Soluble Fiber Supplementation Reduces Risk of Gestational Diabetes in Women with Obesity in a Randomized Controlled Trial	Determine the effect of blueberry and soluble fiber supplementation on GDM risk and GWG for pregnant people with obesity	34	~18 weeks	prenatal	prenatal	Overweight/obesity; Gestational diabetes	Diet	Reduced GWG; lower diastolic blood pressure	Clinic	No	Race: African American and Hispanic	yes	obese
Bublitz 2023	Feasibility, acceptability, and preliminary effects of mindfulness training on antenatal blood pressure	Test the feasibility, acceptability, and effects of minfulness training on rates of HDP among pregnant women at risk for HDP	29	8 weeks	prenatal	prenatal	Hypertensive disorders of pregnancy	Other: mindfulness training	Feasibility; improved blood pressure	Clinic	No	Only provided White race and Hispanic ethnicity	no	none
Cahill 2018	Weight Control Program and Gestational Weight Gain in Disadvantaged Women with Overweight or Obesity: a Randomized Clinical Trial	Evaluate whether an optimized, home-based, lifestyle weight management program could affect GWG and improve maternal and neonatal outcomes in socioeconomically disadvantaged African American pregnant people with overweight or obesity at the beginning of pregnancy.	267	20 weeks (10 biweekly home visits during pregnancy)	prenatal	prenatal	Overweight/obesity	Diet; Physical activity	Reduced GWG	Clinic	Yes	Only Black/African American race reported since this was conducted in this population	yes	overweight or obese
Downs 2017	Randomized face-to-face vs. home exercise interventions in pregnant women with gestational diabetes	Evaluate the effects of a semi-intensive, face-to-face exercise intervention and a minimum-contact, home-based exercise intervention compared to the standard of care (control) on exercise behavior, its motivational determinants, blood glucose levels, and insulin use of women with GDM.	41	12 weeks	prenatal	prenatal	Gestational diabetes	Diet; Physical activity	Reduced blood glucose levels	Clinic	No	Race and ethnicity: non-Hispanic White, Asian American, Hispanic, American Indian, Other	no	none
Downs 2021	Adaptive, behavioral intervention impact on weight gain, physical activity, energy intake, and motivational determinants: results of a feasibility trial in pregnant women with overweight/obesity	Reduce GWG in pregnant people with overweight/obesity through mHealth targeted to diet and physical activity	31	8-12 weeks through 36 weeks	prenatal	prenatal	Overweight/obesity	Diet; Physical activity	Intervention feasible	Social media or online	No	Race: Caucasian/white , Asian	yes	overweight or obese
Durnwald 2016	A Randomized Clinical Trial of an Intensive Behavior Education Program in Gestational Diabetes Mellitus Women Designed to Improve Glucose Levels on the 2-Hour Oral Glucose Tolerance Test	Evaluate whether pregnant people with GDM enrolled in an intensive behavior educational program demonstrate lower mean fasting glucose levels on the 2-hour 75g oral glucose tolerance test at 6-12 weeks postpartum compared to routine GDM management	101	30 weeks through 6-12 weeks postpartum	postpartum	postpartum	Gestational diabetes	Diet; Physical activity	Null	Clinic	Yes	Reported correctly	yes	none
Ferrara 2011	A pregnancy and postpartum lifestyle intervention in women with gestational diabetes mellitus reduces diabetes risk factors: a feasibility randomized control trial	Evaluate feasibility of a lifestyle intervention initiated soon after GDM diagnosis and continuing postpartum. The intervention goals were 1) help postpartum people return to prepregnancy weight or 5% below for postpartum people with overweight/obesity	197	GDM diagnosis through 1 year postpartum	postpartum	postpartum	Gestational diabetes	Diet; Physical activity	Decreased dietary fat intake	Clinic	No	Reported correctly	yes	none
Ferrara 2020	A telehealth lifestyle intervention to reduce excess gestational weight gain in pregnant women with overweight or obesity (GLOW): a randomised, parallel-group, controlled trial	Reduce excess GWG through a behavioral lifestyle intervention adapted from the Diabetes Prevention Program	394	8-15 weeks through 29-38 weeks	prenatal	prenatal	Overweight/obesity	Diet; Physical activity; Other: stress management	Reduced GWG	Clinic	No	Race and ethnicity: Asian, White, Hispanic, African American, Multiracial/Other	yes	overweight or obese

Gallagher 2018	Greater Neonatal Fat-Free Mass and Similar Fat Mass Following a Randomized Trial to Control Excess Gestational Weight Gain	Determine the effectiveness of controlling GWG in the second and third trimesters on neonate body composition	210	15 weeks through delivery	prenatal	prenatal	Overweight/obesity	Diet; Physical activity; Other: counseling/coaching	Reduced GWG	Clinic	Yes	Race: White, Black, Other, More than One, Unknown	no	overweight or obese
Gibbs 2024	The sedentary behavior reduction in pregnancy intervention (SPRING) pilot and feasibility randomized trial	To demonstrate feasibility, acceptability, and initial efficacy of a lower intensity intervention targeting reduced sedentary behavior and increased standing and steps	51	Clinic and online	prenatal	prenatal	High risk for any APO, high sedentary behavior	Physical activity and Other: counseling/coaching	Reduced sedentary time	Clinic and online	Yes	Overall hispanic yes/no, but no ethnicity data provided for individual race data	yes	none
Goletzke 2021	Effect of a Low-Glycemic Load Diet Intervention on Maternal and Pregnancy Outcomes in Obese Pregnant Women	Compare effects of low vs high glycemic diets during the second half of pregnancy on maternal glucose metabolism and pregnancy outcomes in women with overweight or obesity	109	20 weeks through delivery	prenatal	prenatal	Overweight/obesity	Diet	Reduced GWG	Clinic	No	Listed all as ethnicities: Hispanic, African American, Caucasian, Asian, Mixed Ethnicity, Hawaiian/Pacific Islander	yes	obese
Hawkins 2015	A pregnancy lifestyle intervention to prevent gestational diabetes risk factors in overweight Hispanic women: a feasibility randomized controlled trial	Evaluate the feasibility of a lifestyle intervention among Hispanic pregnant people with overweight or obesity who have a higher risk of GDM. The aims of the intervention were to reduce excess gestational weight gain, increase postpartum weight loss and improve maternal metabolic status in this population.	68	~26 weeks	prenatal	postpartum	Overweight/obesity	Diet; Physical activity	Increased minutes of vigorous physical activity	Clinic	Yes	100% Hispanic, unsure if authors asked about identifying with other racial categories	yes	overweight or obese
Hernandez 2016	Women With Gestational Diabetes Mellitus Randomized to a Higher-Complex Carbohydrate/Low-Fat Diet Manifest Lower Adipose Tissue Insulin Resistance, Inflammation, Glucose, and Free Fatty Acids: a Pilot Study	Determine if a high-complex carb/lower fat diet improves maternal insulin resistance, adipose tissue lipolysis and infant adiposity	12	30-31 weeks until delivery	prenatal	prenatal	Overweight/obesity; Gestational diabetes	Diet	Reduced fasting glucose; reduced free fatty acids	Clinic	No	Race: White, Asian, Hispanic	yes	overweight or obese
Hernandez 2023	Randomization to a Provided Higher-Complex-Carbohydrate Versus Conventional Diet in Gestational Diabetes Mellitus Results in Similar Newborn Adiposity	Determine if following a CHOICE diet compared to a conventional diet would be associated with improved insulin resistance and 24-hr gylcemia in pregnant participants with GDM	46	30-31 weeks through delivery	prenatal	prenatal	Overweight/obesity; Gestational diabetes	Diet; Physical activity	Null	Clinic	No	Overall hispanic yes/no, but no ethnicity data provided for individual race data	yes	overweight or obese
Herring 2016	Preventing excessive gestational weight gain among African American women: a randomized clinical trial	Determine whether a technology-based behavioral weight control intervention would be effective among low-income African American pregnant people with overweight or obesity for decreasing excess GWG	66	20 weeks through delivery	prenatal	prenatal	Overweight/obesity	Diet; Physical activity; Other: self-weighing	Reduced GWG	Clinic	Yes	Only recruited self-identified African-American participants, unsure if authors asked about identifying with Hispanic ethnicity	no	overweight or obese
Holmes 2020	Effect of a short message service intervention on excessive gestational weight gain in a low-income population: a randomized controlled trial	Assess the effectiveness of an 18-week SMS intervention promoting nutrition and physical activity delivered to a low-income population of predominantly pregnant people with overweight/obese in Hawaii on reducing excessive GWG	83	18 weeks	prenatal	prenatal	Overweight/obesity	Diet; Physical activity	Null	Clinic	No	Race and ethnicity: Asian, American Indian, Black, Hispanic, Native Hawaiian, Pacific Islander, White and they note that 45/82 (54.2%) of participants identified with multiple races and ethnicities	no	20-45
Homko 2012	Impact of a telemedicine system with automated reminders on outcomes in women with gestational diabetes mellitus	Examine the impact of an enhanced telemedicine system on glucose control and pregnancy outcomes in pregnant people with GDM	80	GDM diagnosis through delivery (no later than 33 weeks for enrollment)	prenatal	prenatal	Gestational diabetes	Other: diabetes self management (self-monitoring glucose)	Contact between participants with GDM and their providers increased (intervention feasible)	Clinic	No	Race and Ethnicity: White, African American, Latino/Hispanic, Asian and Other	yes	none

Hull 2020	The effect of high dietary fiber intake on gestational weight gain, fat accrual, and postpartum weight retention: a randomized clinical trial	Assess effectiveness of a single goal high fiber diet intervention to prevent excessive GWG compared to usual care	20	12 weeks	prenatal	postpartum	Overweight/obesity	Diet	Reduced GWG; reduced PPWR	Clinic	No	Only 2 categories, Hispanic/Latino and non-Hispanic/Latino	yes	≥22
Jaworsky 2023	Effects of an Eating Pattern Including Colorful Fruits and Vegetables on Management of Gestational Diabetes: A Randomized Controlled Trial	Determine if increasing the consumption of berries and green leafy vegetables, as well as increasing postprandial physical activity, would improve cardiometabolic profiles in pregnant women with GDM.	38	12 weeks	prenatal	prenatal	Gestational diabetes	Diet; Physical activity	Improved blood glucose, decreased serum IL-6, improved HDL cholesterol	Clinic	No	Only ethnicity provided (100% Hispanic)	no	none
Kong 2014	A Pilot Walking Program Promotes Moderate-Intensity Physical Activity during Pregnancy	Determine if a walking intervention in pregnancy reduced PPWR in pregnant people with overweight/obesity. Additionally, determine if previously nonexercising pregnant people with overweight or obesity could increase moderate intensity PA participation via a walking intervention and if this intervention improved pregnancy and birth outcomes	47	15-35 weeks	prenatal	postpartum	Overweight/obesity	Physical activity	Increased minutes of walking	Social media or online	No	Only White reported	yes	overweight or obese
Landon 2009	A multicenter, randomized trial of treatment for mild gestational diabetes	Determine if treatment of mild GDM reduces perinatal and obstetrical complications	958	~24 weeks through delivery	prenatal	prenatal	Gestational diabetes	Diet; Other: self-monitoring blood glucose	Reduced GWG; lower rates of HDP; GDM controlled	Clinic	No	Black, White, Asian, Hispanic, Other	yes	none
LeBlanc 2021	Weight loss prior to pregnancy and subsequent gestational weight gain: Prepare, a randomized clinical trial	Determine whether prepregnancy weight loss reduces GWG and improves pregnancy outcomes	169	prepregnancy (up to 2 years) through delivery	prenatal	prenatal	Overweight/obesity	Diet; Physical activity	Null	Social media or online	Yes	Race Categories: White, Asian, Black, More than One, Did not Report	no	≥27
Liu 2021	A Behavioral Lifestyle Intervention to Limit Gestational Weight Gain in Pregnant Women with Overweight and Obesity	Examine the impact of a pregnancy and postpartum behavioral lifestyle intervention (vs. standard care) on postpartum weight retention during the first year after delivery among white and African American women with overweight or obesity.	219	≤18 weeks through delivery	prenatal	prenatal	Overweight/obesity	Diet; Physical activity; self-monitoring	Reduced rates of HDP	Social media or online	No	White, Black/African American (recruited to reflect South Carolina's population)	no	overweight or obese
Mackeen 2022	Encouraging appropriate gestational weight gain in high-risk gravida: a randomized controlled trial	Decrease excessive GWG among women with pregestational obesity	224	<17 weeks through delivery	prenatal	prenatal	Overweight/obesity	Diet; Physical activity	Reduced GWG for participants with class III obesity	Clinic	No	91.8% intervention and 86% control are non-Hispanic white. Other races and ethnicities not reported	no	obese
Nobles 2015	Effect of an exercise intervention on gestational diabetes mellitus: a randomized controlled trial	Examine the effect of a prenatal exercise intervention on GDM	241	12 weeks	prenatal	prenatal	Overweight/obesity; Gestational diabetes	Physical activity	Null	Clinic	No	Only Hispanic and Non-Hispanic categories reported	yes	overweight or obese
Osmundson 2016	Early Screening and Treatment of Women with Prediabetes: a Randomized Controlled Trial	Examine whether treatment of pregnant people with a first-trimester A1C of 5.7 to 6.4% reduces the incidence of GDM compared with usual prenatal care.	83	first trimester through delivery	prenatal	prenatal	Gestational diabetes	Diet; Other: Self-monitoring	Reduced GDM rates for participants without obesity; lower A1c levels	Clinic	No	Race: White, Black, Hispanic, Asian	yes	none
Palnati 2021	The Impact of a Lifestyle Intervention on Postpartum Weight Retention Among At-Risk Hispanic Women	Examine the effect of a lifestyle intervention on postpartum weight retention in Hispanic pregnant people with abnormal glucose tolerance in their current pregnancy.	204	29 weeks through 1 year postpartum	postpartum	postpartum	Gestational diabetes	Diet; Physical activity	Reduced PPWR	Clinic	Yes	100% Hispanic participants "Hispanic ethnicity was identified via self-report in the manner of the U.S. Census."	yes	none
Pauley 2018	Gestational Weight Gain Intervention Impacts Determinants of Healthy Eating and Exercise in Overweight/Obese Pregnant Women	Establish the feasibility of the intervention components and the extent to which the theory of planned behavior and self-regulation constructs motivated pregnant people with overweight/obesity to engage in exercise and healthy eating behaviors.	17	6 week intervention	prenatal	prenatal	Overweight/obesity	Diet; Physical activity	Improved attitudes and self-regulation regarding exercise and nutritious eating	Clinic	No	Ethnicity (Hispanic/non-Hispanic) is separted, but Caucasian is the only race category reported	unknown	overweight or obese

Phelan 2018	Randomized controlled clinical trial of behavioral lifestyle intervention with partial meal replacement to reduce excessive gestational weight gain	Determine the efficacy of a multicomponent behavioral lifestyle intervention with partial meal replacement on GWG rates in Hispanic and non-Hispanic pregnant people with overweight or obesity	257	9-16 weeks through delivery	prenatal	prenatal	Overweight/obesity	Diet; Physical activity	Reduced GWG; lower blood trigylceride levels	Clinic	Yes	Ethnicity (Hispanic/non-Hispanic) is separated, races are American Indian, Alaskan Native, Asian, Black or African American, Native Hawaiian or Pacific Islander, White, and Other. Participants could select multiple races/ethnicities	yes	overweight or obese
Phillips 2019	Combined financial incentives and behavioral weight management to enhance adherence with gestational weight gain guidelines: a randomized controlled trial	Determine the combined efficacy of financial incentives and behavioral weight management on adherence to GWG guidelines in pregnant people with overweight or obesity	124	16 weeks through delivery	prenatal	postpartum	Overweight/obesity	Diet; Other: counseling/coaching	Null	Clinic	No	White is only category reported	no	overweight or obese
Reader 2006	Impact of gestational diabetes mellitus nutrition practice guidelines implemented by registered dietitians on pregnancy outcomes	Determine if nutrition care delivered by RDs using nutrition practice guidelines for GDM results in different care and better pregnancy outcomes compared with usual nutrition care provided by RDs	215	GDM diagnosis through delivery (approximately 9 weeks)	prenatal	prenatal	Gestational diabetes	Diet	Less insulin required for GDM treatment	Clinic	No	Ethnicity: White, Hispanic, African American, Other	yes	none
Redman 2017	Effectiveness of SmartMoms, a Novel eHealth Intervention for Management of Gestational Weight Gain: Randomized Controlled Pilot Trial	Decrease the proportion of pregnant people who exceed the IOM GWG guidelines	54	13 wks 5 days through delivery	prenatal	postpartum	Overweight/obesity	Diet; Physical activity	Reduced GWG	Clinic	No	Black, White, and Other are the only categories reported. No ethnicity data reported	yes	overweight or obese
Rhodes 2010	Effects of a low-glycemic load diet in overweight and obese pregnant women: a pilot randomized controlled trial	Examine the effects of a low glycemic load diet in overweight and obese pregnant people with the provision of specific foods to enhance treatment fidelity.	46	13-28 weeks through delivery	prenatal	prenatal	Overweight/obesity	Diet	Reduced blood levels of triglycerides, total cholesterol and C-reactive protein	Clinic	No	Race: non-Hispanic white, non-Hispanic Black, Hispanic, Asian, More than one, Missing, Other	yes	overweight or obese
Sacks 2006	Managing type I diabetes in pregnancy: how near normal is necessary?	This was a feasibility study to determine if pregnant people with type I diabetes managed with liberal target glucose values will have a decreased frequency of hypoglycemia with no differences in adverse outcomes compared with tightly controlled subjects.	22	<13 weeks gestation through delivery	prenatal	prenatal	Type 1 diabetes	Diet; Other: glucose self-monitoring, counseling/coaching	Improved glucose levels; reduced hypoglycemia	Clinic	No	Race: Caucasian, Other	unknown	none
Sugino 2022	A maternal higher-complex carbohydrate diet increases bifidobacteria and alters early life acquisition of the infant microbiome in women with gestational diabetes mellitus	Determine if consumption of a higher complex carbohydrate/lower fat diet would favorably alter maternal and infant microbiome composition compared to a conventional diet in pregnant people with GDM	34	30-31 weeks through delivery	prenatal	prenatal	Overweight/obesity; Gestational diabetes	Diet	Improved maternal gut microbiome	Clinic	No	Caucasian, Asian, Native American or Alaska Indian, Other	yes	overweight or obese
Thomas 2022	A Web-Based mHealth Intervention With Telephone Support to Increase Physical Activity Among Pregnant Patients With Overweight or Obesity: Feasibility Randomized Controlled Trial	Test the acceptability and feasibility of a pilot mHealth lifestyle intervention for pregnant patients with overweight or obesity to promote moderate intense physical activity, encourage guideline-concordant GWG, and inform the design of a larger pragmatic cluster randomized controlled trial.	68	8-15 weeks through delivery	prenatal	prenatal	Overweight/obesity	Diet; Physical activity	Improved self monitoring of weight; increased physical activity levels	Clinic	Yes	Reported correctly	yes	overweight or obese

Thornton 2009	Perinatal outcomes in nutritionally monitored obese pregnant women: a randomized clinical trial	(1) Compare perinatal outcomes of pregnant people with obesity treated in the conventional manner compared to nutritional monitoring; (2) Determine the effects of weight stabilization in pregnant people with obesity on perinatal morbidity and birth weight of newborns; (3) Determine perinatal differences in the study group's adherence vs non-adherence to a prescribed nutritional regimen applicable to the general practice of obstetrics; (4) Evaluate perinatal outcomes of pregnant people with obesity who had gained 15 pounds or more during their pregnancy compared to those who gained fewer than 15 pounds, irrespective of whether they were in the control or study group; and (5) Evaluate perinatal outcomes of pregnant people with obesity who gained fewer than 10 pounds during pregnancy compared to those who gained 10 pounds or more, irrespective of whether they were in the control or study group.	232	12-28 weeks through delivery	prenatal	postpartum	Overweight/obesity	Diet	Reduced GWG; lower rates of HDP	Clinic	No	Caucasian, Indian, Latina, African American	yes	obese
Trout 2016	Macronutrient composition or social determinants? Impact on infant outcomes with gestational diabetes mellitus	Evaluate at 2 socioeconomically diverse sites: 1) effects of a maternal carbohydrate-restricted diet vs usual pregnancy diet on maternal outcomes of blood glucose, weight gain, common medical comorbidities, and incidence of medical procedures and 2) effects of a maternal carbohydrate restricted diet versus usual pregnancy diet on infant outcomes of birthweight, macrosomia, and adverse perinatal events	68	24-28 weeks (GDM diagnosis) up to 35 weeks through delivery	prenatal	prenatal	Gestational diabetes	Diet	Null	Clinic	No	Race: White non-Hispanic, Black, Asian, Hispanic	yes	none
Twedt 2023	Sleep intervention and glycemic control in gestational diabetes mellitus: a feasibility study	Determine the feasibility of a sleep education program targeted to pregnant women with GDM	70	GDM diagnosis through 35 weeks	prenatal	prenatal	Gestational diabetes	Other: sleep	Feasibility	Clinic	No	Only race provided: White, Black, Asian, Other	no	none
VanHorn 2018	Dietary Approaches to Stop Hypertension Diet and Activity to Limit Gestational Weight: maternal Offspring Metabolics Family Intervention Trial, a Technology Enhanced Randomized Trial	Investigate whether a calorie-controlled, Dietary Approach to Stop Hypertension (DASH)–type diet and lifestyle intervention guided by a Registered Dietitian Nutritionist (RDN) coach using commercially available weight-loss technology could safely be applied to limit GWG and improve diet quality and physical activity.	281	20 weeks	prenatal	prenatal	Overweight/obesity	Diet; Physical activity	Reduced GWG	Clinic	Yes	Ethnicity (Hispanic/non-Hispanic) is separated, races reported are White, Black or African American, and Other	yes	overweight or obese
Vesco 2014	Efficacy of a group-based dietary intervention for limiting gestational weight gain among obese women: a randomized trial	Determine whether the weight management model often used in nonpregnant adults (weekly, group-based weight management intervention focused on diet and behavior change) would be effective among pregnant people with obesity for limiting GWG and reducing proportion of LGA infants	114	8-21 weeks through delivery	prenatal	postpartum	Overweight/obesity	Diet; Physical activity	Reduced GWG; reduced PPWR	Clinic	No	Only White reported	yes	obese
Yeo 2008	A comparison of walking versus stretching exercises to reduce the incidence of preeclampsia: a randomized clinical trial	Compare a walking exercise to a stretching program during pregnancy in high-risk pregnant people who were sedentary and had previously experienced preeclampsia.	79	~18 weeks through delivery	prenatal	prenatal	Hypertensive disorders of pregnancy	Physical activity	increased transferrin levels	Clinic	No	Only non-Hispanic White reported, everyone else is Other	yes	none
